# Mild cognitive impairment: effect of education on the verbal and nonverbal tasks performance decline

**DOI:** 10.1002/brb3.88

**Published:** 2012-08-22

**Authors:** Konstantinos Vadikolias, Anna Tsiakiri-Vatamidis, Grigorios Tripsianis, Georgios Tsivgoulis, Panagiotis Ioannidis, Aspasia Serdari, John Heliopoulos, Miltos Livaditis, Charitomeni Piperidou

**Affiliations:** 1 Department of Neurology, Democritus University of Thrace, University Hospital of AlexandroupolisGreece; 2 Department of Medical Statistics, Democritus University of ThraceGreece; 3 Second Department of Neurology, Aristotle University of Thessaloniki, AHEPA University HospitalGreece; 4 Department of Psychiatry, Democritus University of Thrace, University Hospital of AlexandroupolisGreece; 5 International Clinical Research Center, St. Anne's University Hospital BrnoBrno, Czech Republic

**Keywords:** Cognitive reserve, mild cognitive impairment, nonverbal, verbal

## Abstract

We sought to longitudinally evaluate the potential association of educational level with performance on verbal and nonverbal tasks in individuals with mild cognitive impairment (MCI). We evaluated patients with MCI, age >50 years, no medication intake, absent vascular risk factors, and no lesions on brain magnetic resonance imaging (MRI). Each patient underwent a clinical assessment packet and a series of neuropsychological tests of the language and constructional praxis subtests of Cambridge Cognitive Examination (CAMGOG) and the Boston naming test (BNT), at baseline, 6 months, and 12 months. Educational levels were defined taking into account the total years of education, the school level, and diplomas. MCI patients with low education level showed a stepwise reduction in scores of naming objects (NO; *P* = 0.009), definition (DF; *P* = 0.012), language (LT; *P* = 0.021), constructional praxis (CD; *P* = 0.022), confrontation naming skills (BXB; *P* = 0.033), phonemic help (BFB; *P* = 0.041), and BNT (*P* = 0.002). Analysis of covariance, controlling for baseline scores, showed that education was associated with NO score (*P* = 0.002), DF score (*P* = 0.005), LT (*P* = 0.008), CD score (*P* = 0.008), BXB score (44.36 ± 1.84, *P* = 0.0001), BFB (*P* = 0.022), and BNT (*P* = 0.004). Our findings indicate that education appeared to affect verbal and nonverbal task performance in MCI patients. Despite the fact that higher educated patients are more acquainted with the tasks, slower deterioration in consecutive follow-up examinations could be explained by the cognitive reserve theory. The potential association of this protective effect with delayed onset of symptoms deserves further investigation.

## Introduction

Education is considered to provide a cognitive and neurological reserve through neuronal changes or increased efficacy of processing networks. The “reserve” hypothesis suggests that education should affect the clinical expression of Alzheimer's disease (AD). The concept of cognitive reserve has been proposed to account for the disjunction between the degree of brain damage or pathology and its clinical manifestations (Stern 2009). Twenty-five percent of elders whose neuropsychological testing is unimpaired prior to death meet full pathologic criteria for AD (Ince 2001), suggesting that this degree of pathology does not invariably result in clinical dementia. Educational and occupational exposure and leisure activities are considered that as related with a reduced risk of developing dementia (Stern 2009). Neuropathologic correlations support this theory showing that individuals with greater cognitive reserve, as reflected in years of education, are better able to cope with AD brain pathology without observable cognitive deficits (Roe et al. 2007). However, results from studies examining the relation of the education level with other than the clinical onset aspects, such as the rate of cognitive decline, were not consistent. In a study of AD patients with mild or moderate stage, higher educational attainment was associated with a slower rate of cognitive decline on the Mini-Mental State Exam (MMSE) (Fritsch et al. 2001). Another study showed that higher educational attainment was associated with a slightly accelerated rate of cognitive deterioration (Wilson et al. 2009). Data analysis of a large cohort of participants in the Victoria Longitudinal Study showed that years of education were strongly related to cognitive level in all domains, particularly verbal fluency, but education was not related to rates of change over time for any cognitive domain (Wilson et al. 2004). In a prospective community survey in old subjects without an established clinical diagnosis of AD, education was robustly associated with level of cognitive function but not with the rate of cognitive decline (Zahodne et al. 2011). A meta-analysis of data of 34 previously published studies showed that education, hypertension, objective indices of health, cardiovascular disease, and apolipoprotein E (APOE) were associated with cognitive decline in old-age subjects (Anstey and Christensen 2000).

As mild cognitive impairment (MCI) is a clinically and pathologically heterogeneous state, showing a conversion rate into dementia of 11-33% within 2 years (Gauthier et al. 2006) or approximately 12% per year (Petersen et al. 1999; Anchisi et al. 2005), the question about the appliance of the cognitive reserve theory in MCI has probable conflicting answers. Recent investigations based on neuroimaging measurements (Solé-Padullésa et al. 2009), biochemical methods (Rolstad et al. 2010), and epidemiological studies (Afgin et al. 2012) were indicative that the cognitive reserve hypothesis may be applied also in MCI subjects.

In view of the former considerations, we sought to evaluate whether higher educated subjects with amnestic MCI (aMCI), without systemic diseases, cerebrovascular disease, hypertension, or other vascular risk factors achieve better performance on a series of verbal and nonverbal tasks than lower educated individuals and whether this effect of education persists in a series of repeated examinations over time, supporting the cognitive reserve theory.

## Materials and Method

### Subjects

We evaluated prospectively a cohort of consecutive individuals referred from the Dementia Outpatient Clinic fulfilling the following inclusion criteria: (1) diagnosis of aMCI (Petersen et al. 2001), (2) age 50 years or older, and (3) fluency in Greek language. We excluded subjects with score 13 or higher on the Hamilton Depression Scale (Hamilton 1967) and 12 or higher on the Neuro-Psychiatric Inventory (NPI; Cummings et al. 1994), presence of concomitant neurological or psychiatric disorders or systemic diseases, severe and uncorrected visual or auditory handicaps that would interfere with test performance or cognitive disorders, cognitive decline related to other causes (e.g., hypothyroidism), family history of dementia, clinical or neuroimaging evidence (e.g., silent infarcts or white-matter lesions on brain magnetic resonance imaging [MRI]) of vascular cognitive impairment, vascular risk factors (hypertension, diabetes mellitus, metabolic syndrome, heart disease, current smoking, and hyperlipidemia), and intake of acetylcholinesterase inhibitors (donepezil, rivastigmine, and galantamine), memantine, or other drugs with known direct CNS effects.

This study was approved by the Ethics Committee of our institution. All participants and their caregivers were informed and gave informed consent for taking part in this study.

### Clinical evaluation – neuropsychological tests

Each subject underwent the clinical assessment packet recommended by the Consortium to Establish a Registry for AD (CERAD) (Morris et al. 1989) and a hemi-structural interview. Neurological examination and psychiatric evaluation were performed by a team of experienced neurologists and psychiatrists. Cognitive tests were performed by a neuropsychologist (A.T.). All participants were examined at baseline, 6 months, and 12 months. All the measurements performed by the same examiner over time. Educational level was divided into two categories: (a) low: nonhigh school graduates or <6 years of education and high school graduates or maximum 15 years of education, (b) high: college/university or professional school graduates or >15 years of education.

As an overall measure for cognitive impairment, we used the MMSE (Folstein et al. 1975). We selected neuropsychological tests primary reflecting verbal and nonverbal functions. Verbal tests included the language subtest of Cambridge Cognitive Examination (CAMCOG) (Huppert et al. 1995, 1996). CAMCOG is designed to assess the range of cognitive functions required for a diagnosis of dementia, and to detect mild degrees of cognitive impairment which assesses naming objects (NO score: 0–14), comprehension (UN score: 0–7), definition (DF score: 0–6), repetition (RP score: 0–1), language (LT score: 0–28), and abstractive thought (AT score: 0–8). Boston naming test (BNT) (Kaplan et al. 1983) was also included in verbal assessment examining confrontation naming skills without help (BXB), semantic help (BSB), phonemic help (BFB), and time needed to complete the task (BT). Nonverbal tests comprised the constructional praxis subtest of CAMCOG examining copying and drawing (CD score: 0–6), spontaneous writing (SW score: 0–1), ideational praxis (IP score: 0–5), following commands (FC score: 0–4), and writing (WR score: 0–2) (score 0 indicates a poor performance).

### Statistical analyses

Statistical analyses were performed using the Statistical Package for the Social Sciences (SPSS), version 19.0 (SPSS, Inc., Chicago, IL). The normality of continuous variables was tested with Kolmogorov–Smirnov test. Continuous variables were expressed as mean ± standard deviation (SD), and categorical variables were expressed as frequencies and percentages (%). The chi-square test and Student's *t*-test were used to evaluate differences in patients' characteristics between patients with low and high education level. Repeated measures analysis of variance (ANOVA) was used to examine the changes of the scores of cognitive function tests throughout the follow-up time; post hoc analysis was performed using Bonferroni's correction for multiple comparisons. The interaction between levels of education and the change of cognitive function tests over time was established by two-way analysis of variance. Linear regression analysis and analysis of covariance (ANCOVA) were performed to investigate the effect of education on the cognitive function tests on the 12th month, adjusting for baseline scores. Correlation calculations between education (in years) and the changes of the scores of cognitive function tests were performed by Pearson's correlation coefficient (*r*). All tests were two tailed, and statistical significance was considered for *P*-values less than 0.05.

## Results

A total of 32 patients with aMCI (mean age 68.81 ± 8.40 years, 65.6% men) met the inclusion criteria. MMSE score was 27.88 ± 1.62. Years of education ranged from 0 to 16, with a median value of 12 years; patients were divided into following two educational levels: low level (*n* = 18) and high level (*n* = 14). The two educational groups did not differ in terms of gender (61.1% men vs. 71.4% men, *P* = 0.542), age (69.17 ± 9.10 years vs. 68.36 ± 8.50 years, *P* = 0.799), disease duration >2 years (33.3% vs. 42.9%, *P* = 0.581), and MMSE score (27.39 ± 1.61 vs. 28.53 ± 1.66, *P* = 0.060). Two subjects (low education level group) fulfilled the criteria of AD at the last 12-month assessment.

Scores of all cognitive function tests at baseline, 6 months, and 12 months in relation to the education level are shown in Tables 1–3. Within MCI patients with low education level, one-way repeated measures ANOVA showed a progressive reduction over time of the performance in the following tests: NO (*P* = 0.001), DF (*P* = 0.021), LT (*P* = 0.006), AT (*P* = 0.019), CD (*P* = 0.018), BXB (*P* = 0.011), and BNT (*P* = 0.001); a tendency toward lower scores over time were also observed in the BFB (*P* = 0.080) and ideational praxis score (*P* = 0.061). On the contrary, none of the tests changed significantly over time within MCI patients with high education level. Education influenced the performance over the follow-up time of seven of the above function tests, as the two-way mixed ANOVA showed that the interaction between the levels of education and the change over time was statistically significant for NO (*P* = 0.009), DF (*P* = 0.012), LT (*P* = 0.021), CD (*P* = 0.022), BXB (*P* = 0.033), BFB (*P* = 0.041), and BNT (*P* = 0.002) (Tables 1–3).

**Table 1:** Verbal scores of subjects with MCI in relation to their educational level

	Verbal scores (mean values ± SD)				
	
	Baseline	6th month	12th month	*P*-value	ΔScore_0_12_
Naming objects score				0.009^2^	
Low education level	10.33 ± 1.37	10.22 ± 1.52	9.28 ± 1.87	0.001^1^	−1.05 ± 1.26
High education level	12.00 ± 1.46	12.22 ± 1.30	12.36 ± 1.08	0.712^1^	0.36 ± 0.68^3^
Comprehension score				0.822^2^	
Low education level	6.00 ± 0.77	5.89 ± 0.67	5.66 ± 0.97	0.350^1^	−0.33 ± 1.24
High education level	6.36 ± 0.73	6.43 ± 0.46	6.36 ± 0.70	0.691^1^	0.00 ± 0.00
Definition score				0.012^2^	
Low education level	4.83 ± 0.78	4.78 ± 0.80	4.33 ± 1.02	0.021^1^	−0.50 ± 0.86
High education level	5.43 ± 0.82	5.36 ± 0.75	5.65 ± 0.52	0.273^1^	0.22 ± 0.46^3^
Repetition score				0.430^2^	
Low education level	0.50 ± 0.51	0.67 ± 0.49	0.61 ± 0.50	0.176^1^	0.11 ± 0.47
High education level	1.00 ± 0.00	1.00 ± 0.00	0.93 ± 0.31	0.577^1^	−0.07 ± 0.31
Language score				0.021^2^	
Low education level	21.66 ± 2.28	21.56 ± 2.15	19.89 ± 3.34	0.006^1^	−1.78 ± 2.88
High education level	24.79 ± 1.91	25.00 ± 2.06	25.29 ± 1.75	0.694^1^	0.50 ± 0.90^3^
Abstractive thought				0.703^2^	
Low education level	4.50 ± 1.62	4.88 ± 1.65	4.05 ± 1.69	0.019^1^	−0.44 ± 1.10
High education level	6.07 ± 1.06	6.21 ± 1.12	6.00 ± 1.30	0.788^1^	−0.07 ± 0.66

ΔScore_0_12_, 12-month change in verbal scores; MCI, mild cognitive impairment.

Statistical significance: ^1^time effect within the same level of education group and ^2^interaction education level × time effect; ^3^statistically significant difference compared with low education level.

**Table 2:** Nonverbal scores of subjects with MCI in relation to their educational level

	Nonverbal scores (mean values ± SD)				
	
	Baseline	6th month	12th month	*P*-value^1^	ΔScore_0_12_
Drawing score				0.022^3^	
Low education level	4.28 ± 0.75	4.44 ± 0.92	3.61 ± 0.85	0.018^1^	−0.67 ± 1.08
High education level	4.78 ± 0.60	4.86 ± 0.61	4.79 ± 0.72	0.763^1^	0.01 ± 0.49^3^
Spontaneous writing score				0.747^3^	
Low education level	1.06 ± 0.24	1.06 ± 0.42	0.94 ± 0.42	0.462^1^	−0.12 ± 0.47
High education level	1.00 ± 0.00	1.00 ± 0.00	1.00 ± 0.00	1.000^1^	0.00 ± 0.00
Ideational praxis score				0.511^3^	
Low education level	4.55 ± 0.51	4.44 ± 0.51	4.11 ± 0.83	0.061^1^	−0.44 ± 0.86
High education level	4.71 ± 0.52	4.71 ± 0.48	4.64 ± 0.59	0.863^1^	−0.07 ± 0.25
Following commands score				0.715^3^	
Low education level	3.94 ± 0.24	3.89 ± 0.32	3.61 ± 0.78	0.126^1^	−0.33 ± 0.84
High education level	4.00 ± 0.00	3.86 ± 0.37	3.78 ± 0.43	0.239^1^	−0.22 ± 0.44
Writing score				0.914^3^	
Low education level	1.72 ± 0.46	1.78 ± 0.43	1.67 ± 0.59	0.485^1^	−0.05 ± 0.42
High education level	1.78 ± 0.44	1.85 ± 0.40	1.78 ± 0.47	0.839^1^	0.00 ± 0.00

ΔScore_0_12_, 12-month change in nonverbal scores; MCI, mild cognitive impairment.

Statistical significance: ^1^time effect within the same level of education group and ^2^interaction education level × time effect; ^3^statistically significant difference compared with low education level.

**Table 3:** Boston Naming Test scores of subjects with MCI in relation to their educational level

	Boston Naming Test scores (mean values ± SD)				
	
	Baseline	6th month	12th month	*P-*value^1^	ΔScore_0_12_
Naming without help score				0.033^3^	
Low education level	33.50 ± 4.84	33.50 ± 5.15	30.55 ± 5.75	0.011^1^	−2.95 ± 5.10
High education level	42.21 ± 4.93	43.00 ± 5.48	43.64 ± 4.48	0.536^1^	1.43 ± 4.03^3^
Naming semantic help score				0.965^3^	
Low education level	4.39 ± 2.52	4.33 ± 2.52	4.33 ± 2.33	0.995^1^	−0.06 ± 3.17
High education level	4.57 ± 1.86	4.22 ± 1.85	4.00 ± 1.66	0.695^1^	−0.57 ± 1.66
Naming phonemic help score				0.041^3^	
Low education level	5.11 ± 2.00	4.61 ± 2.45	4.00 ± 1.64	0.080^1^	−1.11 ± 1.95
High education level	4.08 ± 1.30	4.00 ± 1.52	4.07 ± 1.11	0.809^1^	−0.01 ± 0.54
Boston (BNT) score				0.002^3^	
Low education level	43.00 ± 4.49	42.44 ± 4.18	38.94 ± 4.49	0.001^1^	−4.06 ± 5.74
High education level	51.71 ± 4.18	51.22 ± 4.39	51.72 ± 3.70	0.940^1^	0.01 ± 3.72^3^
Naming time score				0.883^1^	
Low education level	636.4 ± 113.1	658.1 ± 106.0	649.7 ± 136.6	0.527^1^	13.3 ± 102.9
High education level	523.0 ± 108.7	533.6 ± 96.3	526.9 ± 112.8	0.858^1^	3.9 ± 75.1

ΔScore_0_12_, 12-month change in nonverbal scores; MCI, mild cognitive impairment.

Statistical significance: ^1^time effect within the same level of education group and ^2^interaction education level × time effect; ^3^statistically significant difference compared with low education level.

During our sequential evaluations, we considered an outcome of interest, the cognitive performance at our last follow-up evaluation (12 months). Analysis of covariance, controlling for baseline scores, showed a statistically significant effect of education on the NO score (adjusted mean values ± SE, 9.88 ± 0.28 and 11.58 ± 0.44 in the low and high levels of education, respectively, *P* = 0.002), DF score (4.51 ± 0.16 and 5.41 ± 0.27, *P* = 0.005), LT (20.92 ± 0.60 and 23.96 ± 0.93, *P* = 0.008), CD score (3.70 ± 0.19 and 4.68 ± 0.31, *P* = 0.008), BXB score (33.12 ± 1.18 and 44.36 ± 1.84, *P* = 0.0001), BFB (3.62 ± 0.43 and 4.48 ± 0.32, *P* = 0.022), and BNT (41.19 ± 1.39 and 48.84 ± 2.17, *P* = 0.004), with lower scores being documented in the group of patients with low education level. Moreover, similar results were obtained when education was treated as a continuous variable (in years; range, 0–16 years; median value, 6 years); in the linear regression analysis (adjusting for demographic and clinical characteristics and baseline scores), the duration of education was independently and positively associated with the following function tests: NO (*β* = 0.457, SE = 0.087, *P* = 0.001, *R*^2^ = 27.7%), DF (*β* = 0.274, SE = 0.051, *P* = 0.002, *R*^2^=23.8%), LT (*β* = 0.980, SE = 0.141, *P* = 0.014, *R*^2^ = 15.1%), CD (*β* = 0.211, SE = 0.044, *P* = 0.023, *R*^2^ = 12.5%), BXB (*β* = 1.284, SE = 0.267, *P* = 0.017, *R*^2^ = 14.2%), BFB (*β* = 0.204, SE = 0.038, *P* = 0.031, *R*^2^ = 11.9%), and BNT (*β* = 2.085, SE = 0.310, *P* = 0.002, *R*^2^ = 25.3%).

The positive effect of higher education was reflected by comparing the mean change during the 12-month follow-up (ΔScore_0_12_; Tables 1–3) between the two levels of education; statistically significant differences were found on the following function tests: naming objects (NO) (*P* < 0.001), definition (DF) (*P* = 0.008), language (LT) (*P* = 0.008), drawing (CD) (*P* = 0.037), naming without help (BXB) (*P* = 0.013), naming with phonemic help (BFB) (*P* = 0.049), and Boston naming test (BNT) (*P* = 0.029). Finally, statistically significant positive correlations were also found between the duration of education (in years) and the 12-month change (ΔScore_0_12_) of the following function tests: NO (*r* = 0.588, *P* = 0.0004), DF (*r* = 0.487, *P* = 0.005), LT (*r* = 0.522, *P* = 0.002), CD (*r* = 0.408, *P* = 0.020), BXB (*r* = 0.441, *P* = 0.012), BFB (*r* = 0.380, *P* = 0.032), and BNT (*r* = 0.568, *P* = 0.0007).

## Discussion

In this study, higher educational attainment in aMCI subjects was correlated with better performance in verbal and nonverbal tasks during repeated examinations over 1-year period. Subjects with low level of education performed worse than patients with high level of education who presented a more “stable” clinical course. These findings provide support for a cognitive reserve that could alter not only the onset of the symptoms but also the clinical rate slowing the cognitive decline during the predementia phase.

The neurobiologic mechanisms responsible for the association between education and cognitive functions are not known. One plausible explanation is that education impacts the rate at which plaques and tangles accumulate in the brain. Snowdon et al. (1996) found a relation between early life linguistic ability and density of neurofibrillary tangles. In contrast, Del Ser et al. (1999) did not reproduce the former correlation in their autopsy study evaluating patients with AD and Lewy body dementia. In fact, many studies agree that although the education level does not directly impact the accumulation of AD pathology, it can delay the clinical onset of the symptoms (Katzman et al. 1988; Stern et al. 1992a; Stern et al. 1995; Friedland et al. 2001). Alexander et al. (1997), using positron emission tomography, found that premorbid intellectual ability as it estimated by a demographics-based IQ and performance on a measure of word-reading task was inversely correlated with cerebral metabolism in prefrontal, premotor, parietal, and other cerebral regions among patients of similar dementia severity levels and concluded that higher intellectual ability altered the clinical expression of dementia. In other words, a better task performance that is related with higher education seems to mask the clinical expression of a higher degree of neurodegeneration (Bennett et al. 2003; Perneczky et al. 2006; Scarmeas et al. 2006; Stern et al. 1992b).

The potential association of this reserve mechanism with the course of disease in MCI individuals is intriguing and of potential clinical interest. AD pathology seems to progress independently from educational and occupational attainment, and when pathology becomes very severe, there is no longer a substrate for cognitive reserve to come into play (Stern 2002). The results about the rate of cognitive decline in AD patients are inconsistent, supporting a slower decline (Fritsch et al. 2001), no decline (Wilson et al. 2004), or accelerated decline (Teri et al. 1995; Wilson et al. 2000; Wilson et al. 2009; Zahodne et al. 2011) in higher educated subjects. Our results in MCI revealed a slower deterioration in performance of different tasks indicating delay in the cognitive decline in individuals with higher level of education.

Garibotto et al. (2008) showed a significant association between higher education/occupation and lower regional Cerebral Metabolic Rate of glucose consumption (rCMRglc) in posterior temporoparietal cortex and precuneus in AD and aMCI supporting the view that functional reserve is already at play in the MCI, but there are no specific data about the rate of decline in MCI. Karrasch and Laine (2003) showed that the tests of naming, verbal fluency, and verbal memory were affected by educational attainment. Lièvre et al. (2008), using a summary performance-based measure which reflected a range of cognitive abilities, including language and naming, concluded that development of cognitive impairment was highly affected by education. Years of education was also considered the best single predictor of overall cognitive performance (Kaplan et al. 2009) and patients with high education could gain an advantage by being more familiar with the kinds of tasks used in neuropsychological assessments (Kemppainen et al. 2008).

In our study, we found dissociation between verbal and nonverbal patterns. Among the latter, only changes in copying–drawing abilities were related to education. Other studies found no correlation in the nonverbal tasks in AD patients (Filley and Cullum 1997) or in normal elderly subjects (Meguro et al. 2001). In fact, cognitive reserve is not a unitary construct and do not affect all areas of cognitive functioning equally (Stern et al. 1999). In patients with mild AD, the abstract reasoning performance task score was correlated with the years of education (Vliet et al. 2003). Roe et al. (2008) suggest that cognitive reserve, as reflected in education, may have a stronger or earlier effect on specific cognitive processes such as the abstract reasoning, compared with other cognitive processes. An inverse correlation was found in the study by Le Carret et al. (2005).

Indeed, MCI is a clinically heterogeneous state and many factors could alter the tasks performance. In our study, we used very strict inclusion criteria. The participants were free of medications; normal brain MRI without silent infarcts and leucoencephalopathy was a mandatory prerequisite to avoid influences of other factors (Tsivgoulis et al. 2009; Nooyens et al. 2010).

In conclusion, education was found to influence tests performance during follow-up examinations. This effect was present during the 1-year repeated follow-up examinations in a series of verbal and nonverbal tasks supporting a slower decline in higher educated subjects. Our findings are preliminary; inclusion of more subjects and extension of the follow-up assessment beyond the 12 months would be an answer to the difficult question how long this “protective” effect persists.
